# Highly sensitive detection of kojic acid in food samples using fluorescent carbon dots derived from pomegranate peel

**DOI:** 10.1038/s41598-024-70844-2

**Published:** 2024-09-10

**Authors:** Omnia H. Hassan, Ahmed S. Saad, Mohsen Ghali

**Affiliations:** 1 Energy Materials Program, Institute of Basic and Applied Sciences, Egypt-Japan University of Science and Technology, New Borg El-Arab City, Alexandria, 21934 Egypt; 2 PharmD Program, Egypt-Japan University of Science and Technology, New Borg El-Arab 21934, Alexandria, Egypt; 3 Analytical Chemistry Department, Faculty of Pharmacy, Cairo University, Kasr Alaini St. 11562, Cairo, Egypt; 4 Physics Department, Faculty of Science, Kafrelsheikh University, Kafrelsheikh, Egypt

**Keywords:** Carbon dots, Kojic acid, Food safety, Optical sensors, Biomass, Graphene, Nanoscale devices, Nanoscale materials, Other nanotechnology

## Abstract

Kojic acid (KA) has gained significant attention due to its widespread use in the food and cosmetics industries. However, concerns about its potential carcinogenic effects have heightened the need for sensitive detection methods. This study introduces a fluorescence-based optical sensor for the quantification of KA in food samples, utilizing fluorescent carbon dots (CDs) synthesized from pomegranate peel via a hydrothermal method. The Stern–Volmer plot demonstrated a linear response for KA in the range of 120 to 1200 µM, with a Pearson correlation coefficient (r) of 0.9999 and. The sensor exhibited a detection limit of 30 ± 0.04 µM and a limit of quantification (LOQ) of 90 ± 0.14 µM. Application of the developed method to soy sauce and vinegar samples yielded accurate KA determinations, with recoveries of 103.11 ± 0.96% and 104.45 ± 2.15%, respectively. These findings highlight the potential of the proposed sensor for practical applications in food quality and safety assessment, offering valuable insights into the presence of KA in food products.

## Introduction

Kojic acid (KA) is an organic acid with mild strength known by the chemical formula 5-hydroxy-2-(hydroxymethyl)-4-pyrone. It is derived from 4-pyrone, found in various fungi and obtained by aerobic fermentation of microorganisms, most notably *Aspergillus oryzae*, commonly known by its Japanese name Koji^1^. It is extensively utilized worldwide in the production of various foodstuffs, such as soybean paste, soy sauce, and sake. KA growing popularity stems from its effectiveness in multiple applications. It inhibits tyrosinase enzyme in food products, acts as an antioxidant, preserves fermented food from microbial growth, and inhibits nitroso pyrrolidine formation in fried food. Additionally, KA has gained significant attraction in the cosmetic industry due to its well-known skin-whitening effect and its use as a sunscreen. The KA is typically available in the form of creams, serums, and soaps. Metal complexes of kojic acid have the potential to function as radioprotective agents. Furthermore, several derivatives of kojic acid exhibit significant antidiabetic activity by acting as dual agonists for nuclear peroxisome proliferator-activated receptors alpha and gamma. Additionally, kojic acid demonstrates pesticidal properties comparable to those of commercially available pesticides^2–4^.

Allegations have been made regarding the possible carcinogenic, teratogenic, and embryotoxic effects of KA^5^. The hydroxyl group in the KA molecule has the potential to cause free radicals to build up. KA was listed in category III carcinogens in the list of carcinogens published by the WHO in 2017. The Japanese Ministry of Health, Labor, and Welfare re-evaluated the use of KA in the food and personal care industries. Moreover, the US Food and Drug Administration has restricted the use of KA as an over-the-counter pharmaceutical product. Therefore, sensitive detection of KA content is highly important for food safety and product quality control^6,7^. The U.S. Food and Drug Administration indicates that kojic acid can be incorporated at concentrations between 0.1% and 2.0%. In Japan, it has been used for many years at lower concentrations, specifically in meats (0.2%), vegetables (1.0%), and flour (0.1%). Additionally, it is applied in flavorings (0.2%) and syrup products (0.05%). Thus, it is crucial to develop techniques for measuring KA levels in food items and identifying those with greater KA concentrations^4^.

Various techniques have been employed for the detection of KA, including HPLC, capillary electrophoresis^8^, colorimetry^9^, electrochemical^10^, and gravimetric methods. However, the reported methods suffer from several limitations, such as time-consuming pretreatment, expensive equipment, perturbed specificity for KA, or insufficient sensitivity. Therefore, there is a need to develop a simple and effective strategy to detect KA^9^.

Optical sensors encompass a range of nanomaterials, including semiconductor quantum dots, nano-porphyrin, gold nanoparticles, silver nanoparticles, upconversion nanoparticles, nano enzymes, metal oxide nanomaterials, and nanomaterials based on organic fluorescent molecules^11^. The fluorescent sensors that rely on the interaction between a detector and a target analyte have gained significant attention in chemical and biological applications due to their high sensitivity, simplicity, and rapid implementation^12^.

Recently, carbon dots (CDs) have attracted increasing attention owing to excellent photostability and biocompatibility, low toxicity, and easy surface functionalization, which nominate them for a wide range of applications such as bioimaging, sensing, drug delivery, and photocatalysis^13–15^. There are different methods for CDs synthesis, such as hydrothermal, pyrolysis, microwave-assisted, and ultrasonic. The hydrothermal technique has the advantages of cost-effectiveness, easy operation, and simple equipment requirements, and hence has been widely used in the synthesis of CDs^16,17^. The raw materials for CDs synthesis are abundant and categorized into organic and inorganic carbon sources. Organic carbon sources include organic compounds, organic natural products, and biomass waste. Various sources, such as grass, pomegranate peels, household waste, agricultural and livestock residues, as well as forestry leftovers, can provide biomas^18,19^.

Carbon dots synthesized from natural resources have been widely used in various studies as probes to detect different analytes. They have demonstrated the ability to sense heavy metals such as Cu^2+^, Pb^2+^, Co^2+^, Fe^3+^, and Ni^2+^as well as chemical pollutants, pesticides, drugs, and biomolecules. These detection methods have found extensive application in evaluating food quality. The interactions between environmental pollutants and CDs play a crucial role in these applications, leading to fluorescence reduction through quenching or fluorescence enhancement by inhibiting the quenching effect. The mechanisms involved in the quenching of fluorescent CDs in environmental monitoring include dynamic quenching, static quenching (SQE), Förster resonance energy transfer (FRET), inner filter effect (IFE), photoinduced electron transfer (PET), and surface energy transfer (SET)^20–24^. Multiple studies have focused on synthesizing CDs from biomass as a carbon source for sensing purposes. In one such study, Lu et al. used pomelo peel to produce C-dots with a photoluminescence (PL) quantum yield of 6.9%. These CDs were utilized as fluorescent probes to detect Hg^2+^ ions by means of fluorescence quenching. The practical application of this approach was demonstrated by accurately measuring Hg^2+^ concentrations in lake water samples. Another study by Kumar et al. employed the leaves of Ocimum sanctum as a carbon source to synthesize CDs with a PL quantum yield of 9.3%. These C-dots displayed excellent stability in aqueous solutions and were employed as fluorescent sensors to detect Pb^2+^ ions through fluorescence quenching. Successful practical demonstrations were conducted using triple-negative breast cancer cells and real water samples^25,26^.

In this study, a low-cost and environmentally friendly method was employed to synthesize blue luminescent CDs by subjecting powdered pomegranate peels to hydrothermal treatment. Extensive characterization of the CDs was conducted using techniques such as Ultraviolet–visible (UV–vis) absorption spectroscopy, fluorescence spectroscopy, Fourier transform infrared (FT-IR) spectroscopy, X-ray diffraction analysis, and transmission electron microscopy (TEM). The synthesized carbon dots were employed to create a straightforward, sensitive, and reliable optical sensor for detecting kojic acid (KA) in real food samples. This represents the first instance of a carbon dots-based sensor, derived from biomass, metal free being used specifically for the detection of KA. An extensive review of existing literature reveals that our study is the first to utilize carbon dots derived from biomass as an optical sensor for the direct assay of Kojic acid in food products.

## Materials and methods

### Materials

Kojic acid (certified purity 98.5%, Sigma Aldrich), pomegranate peel powder, soy sauce, and vinegar were collected from the local market. Ammonium chloride, sodium chloride, manganous chloride, magnesium chloride, sodium cyanide, sodium sulfate, potassium chloride, cadmium chloride, calcium chloride, sodium nitrite, barium chloride, and acetic acid were obtained from Piochem® Chemicals, Egypt. Cellulose dialysis tubing (1000 Da molecular weight cutoffs) was obtained from Frey Scientific. A Standard solution was prepared using deionized water. A 0.1 M standard aqueous solution for each interferent (${\text{NH}}_{4}^{+}, {\text{Cl}}^{-}, {\text{Na}}^{+}, {\text{Mn}}^{2+}$, ${\text{Mg}}^{2+}$, ${\text{CN}}^{-}$, ${\text{SO}}_{4}^{2-}$, ${\text{K}}^{+}$, ${\text{Cd}}^{2+}$, ${\text{Ca}}^{2+}$, ${\text{NO}}_{2}^{-}$, ${\text{Ba}}^{2+}$, and acetic acid) was prepared.

### Synthesis of fluorescent CDs

**Fig. 1:**
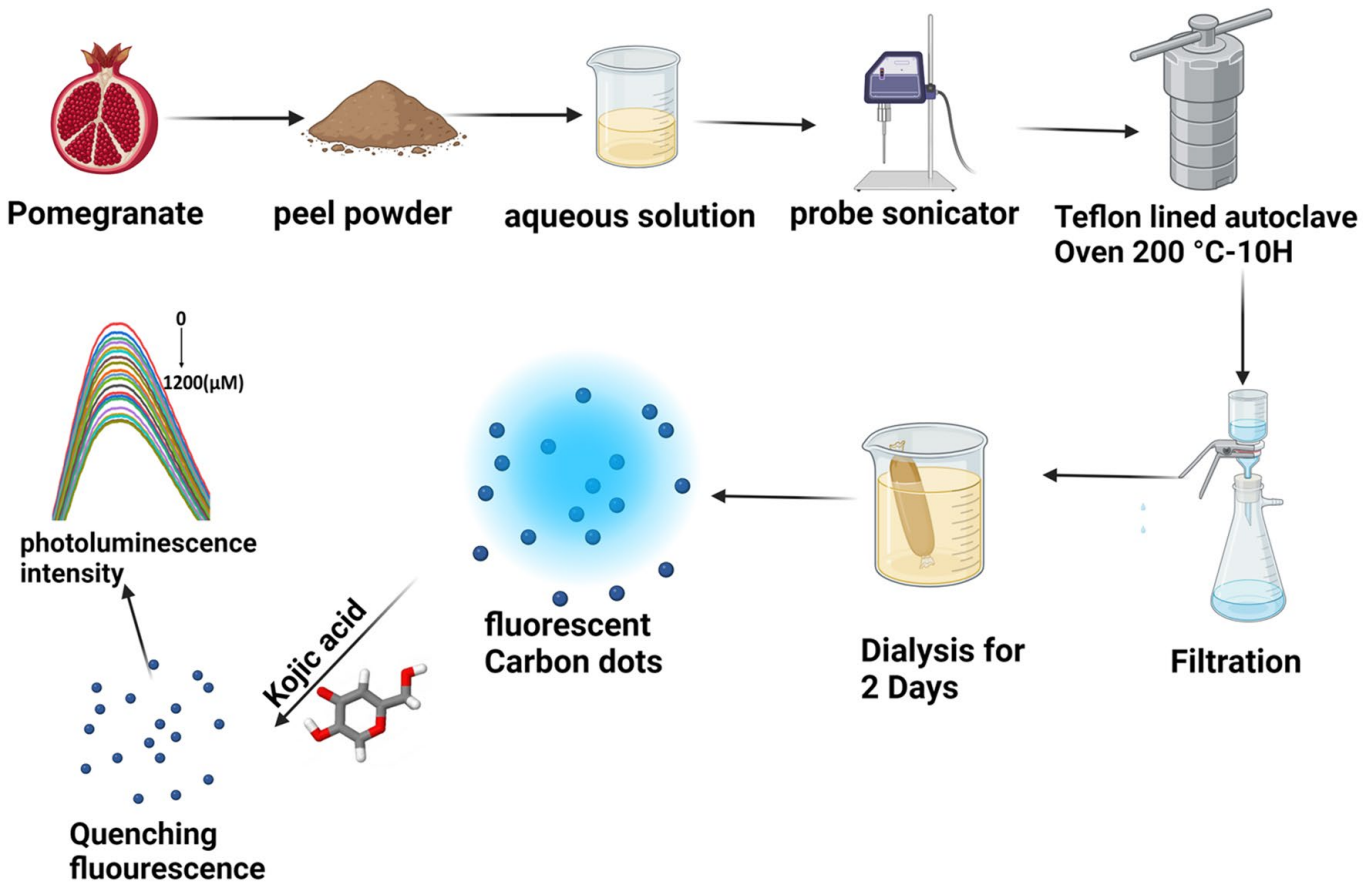
Synthesis of carbon dots from powdered pomegranate peels.

A mass of 300 mg of powdered pomegranate peels was probe-sonicated in 45 ml of ultra-pure water for one hour and transferred to a 750 mL Teflon-lined stainless autoclave. The chamber was sealed and heated to 200 °C for 10 h in the Muffle furnace to complete the hydrothermal reaction, then left to reach room temperature. The product was first filtered through Whatman®-102 filter paper and then through a 0.22 µm membrane filter. The filtrate was dialyzed with a 1 kilo-Dalton cutoff dialysis bag against 200 mL deionized water. The solution was stored in a refrigerator at 4 °C. The process of synthesizing CDs derived from pomegranate peels is shown in Fig. 1.

### Characterization of the synthesized carbon quantum dots

The morphology and mean diameter of the resultant CDs are characterized by a transmission electron microscope (JEOL, JEM-2100F) operating at 200 kV. The X-ray diffraction pattern was measured using X-ray diffractometer (Shimadzu XRD-6100). The resistance to agglomeration and the charge value on the particles was obtained with Zetasizer (Malvern Zetasizer Nano Series). The Fourier transform infrared spectroscopy was measured at wavenumbers ranging from 500 to 4000 using Bruker Optics spectrophotometer (INVENIO S). UV–Vis absorption spectrum was recorded using a Shimadzu UV–Vis spectrophotometer (UV-2600) over the wavelength range from 200 to 800 nm. The photoluminescence full scan for the sample was measured via Shimadzu spectrofluorometer (RF–6000) using different excitation wavelengths in the range of 200–400 nm. The fluorescence intensities were recorded from 380 to 570 nm, while keeping the width of the excitation and emission slits at 10 nm. The fluorescence stability over time was evaluated under various conditions, including exposure to UV and daylight. The lifetime of the CDs was measured with a time-correlated single photon counting lifetime fluorometer.

### Validation of the synthesized CDs for Kojic acid assay

#### Linearity assessment

A blank solution was prepared by diluting 4 mL of CDs working solution in a 5-mL volumetric flask using deionized water. While test solutions of different KA concentrations (from 120 µM up to 1200 µM) were prepared by mixing aliquots of KA standard solution (0.01 M) containing different amounts of KA with 4 mL of CDs working solution in a 5-mL volumetric flask, then completing to the mark using deionized water. The fluorescence intensities of the blank and test solutions were recorded using excitation wavelength of 350.0 nm and 10 nm excitation and emission slit widths. The fluorescence quenching (FQ) was calculated using the following formula:

1 $$FQ={F}_{0}/F$$

where F_0_ and F are the fluorescence intensities of the blank and test solutions, respectively.

#### Selectivity test of KA-CDs

The selectivity test was conducted over two different levels. The first level separately recorded the fluorescence quenching for test solutions prepared by mixing 4 mL CDs working solution, 125 µL of each 0.1 M interferent standard solution, and deionized water to reach the mark in a 5-mL volumetric flask. The prepared solutions included the same CDs concentration with 2500 µM interferent solutions (${\text{NH}}_{4}^{+}, {\text{Cl}}^{-}, {\text{Na}}^{+}, {\text{Mn}}^{2+}$, ${\text{Mg}}^{2+}$, ${\text{CN}}^{-}$, ${\text{SO}}_{4}^{2-}$, ${\text{K}}^{+}$, ${\text{Cd}}^{2+}$, ${\text{Ca}}^{2+}$, ${\text{NO}}_{2}^{-}$, ${\text{Ba}}^{2+}$, and acetic acid). A bar diagram for fluorescence quenching was plotted. The second level separately recorded the fluorescence quenching in solutions containing a mixture of 4 mL CDs working solution, 125 µL of KA standard solution, 125 µL of each 0.1 M interferent standard solution and deionized water to reach the mark in a 5-mL volumetric flask. Each solution included a different interferent at constant concentration of CDs, KA, and interferent (${\text{NH}}_{4}^{+}, {\text{Cl}}^{-}, {\text{Na}}^{+}, {\text{Mn}}^{2+}$, ${\text{Mg}}^{2+}$, ${\text{CN}}^{-}$, ${\text{SO}}_{4}^{2-}$, ${\text{K}}^{+}$, ${\text{Cd}}^{2+}$, ${\text{Ca}}^{2+}$, ${\text{NO}}_{2}^{-}$, ${\text{Ba}}^{2+}$, and acetic acid).

#### Determination of Kojic acid in real samples

The vinegar and soy sauce samples were filtered using a 0.22 μm membrane filter, 20 µL of the filtrate were transferred into a 5-mL volumetric flask, mixed with 4 mL CDs working solution, and deionized water to reach the mark. The standard addition technique was used to determine the concentration of KA in the vinegar sample. The pH of the measured solution was found to be 6.

## Results and discussions

### Structural and optical characterization of CDs

**Fig. 2:**
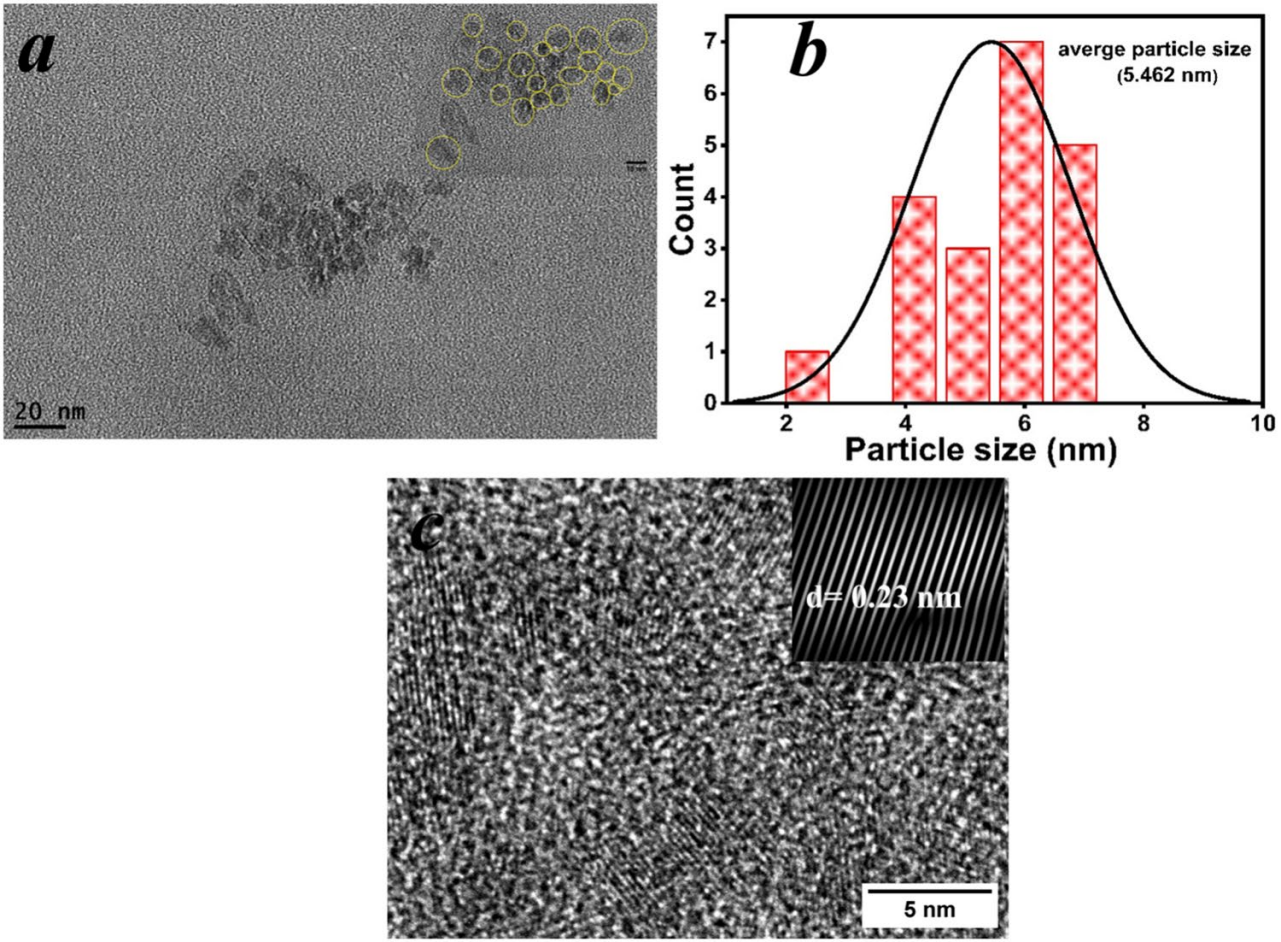
(**a**) Transmission electron microscope image of the synthesized CDs. (**b**) the particle size distribution diagram of the synthesized carbon dots. (**c**) High resolution electron microscope image of the synthesised CDs. The inset shows the d-spacing of the crystallographic planes of the CDs.

**Fig. 3:**
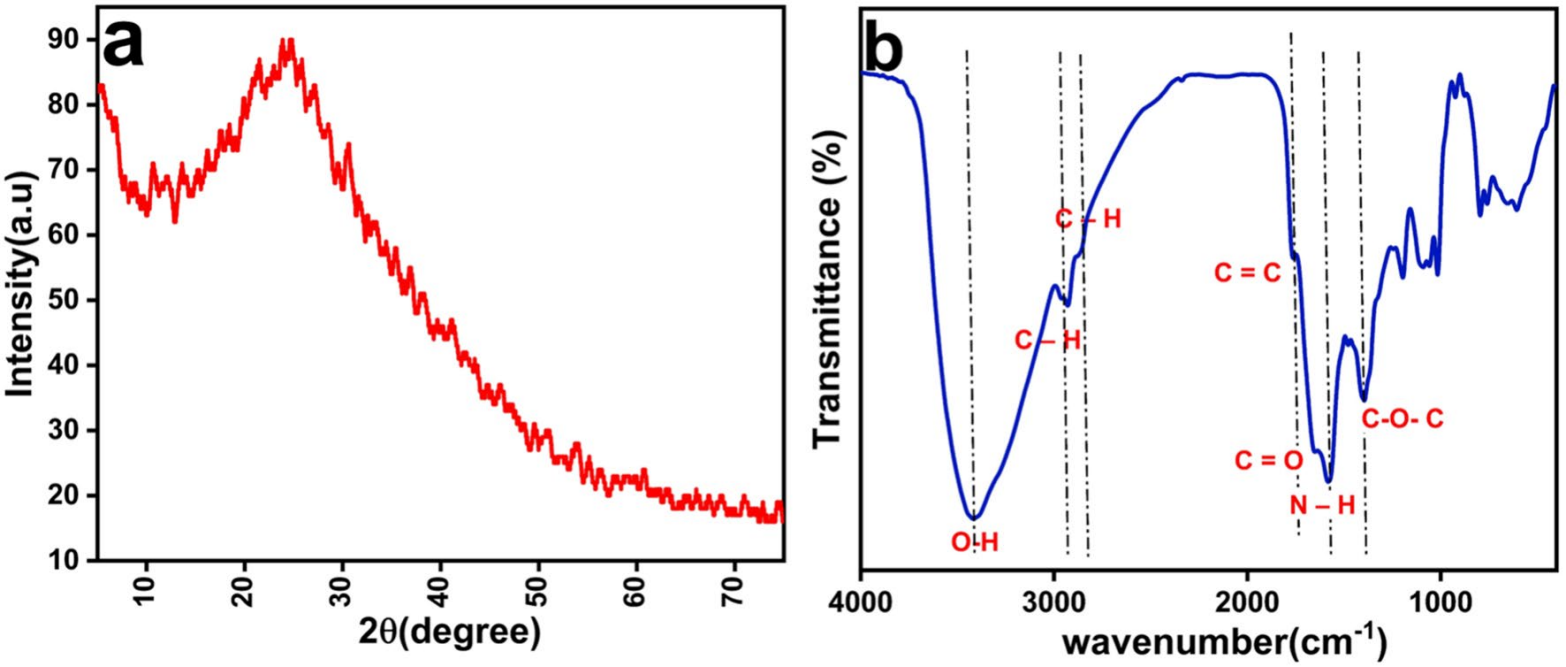
(**a**) X-ray diffraction pattern of the synthesized CDs. (**b**) The FTIR spectrum of synthesized CDs.

Different analytical methods were used to characterize the synthesized carbon dots. The transmission electron microscope (TEM) images showed quasi-spherical particles, as shown in Fig. 2a, with a particle size distribution of 2 nm to 7 nm and an average size of 5.46 nm, Fig. 2b. The high-resolution electron microscope images, Fig. 2c. shows an interplanar distance (d-spacing) of 0.23 nm, both of particle size range which is below 10 nm and the defined interplanar distance confirms the formation of carbon dots^27^. Moreover, we confirm that our carbon dots solution is stable for at least 1 week since the date of the synthesis. While the XRD pattern, Fig. 3a, shows a broad peak at 2θ = 24.9 ͦ, which represents the (002) plan, that confirms the formation of carbon dots^25^. The FTIR spectrum, Fig. 3b, shows a significant broad peak at 3420.1 ${\text{cm}}^{-1}$ corresponding to the symmetric and asymmetric stretching vibration of the hydroxyl group^28^. The peaks at 2926.8 ${\text{cm}}^{-1}$ and 2875.6 ${\text{cm}}^{-1}$ are attributed to the stretching of the C–H bonds^29^. The peak at 1757.3 ${\text{cm}}^{-1}$ is due to the stretching vibration of the C=C bond, while the peak at 1634.3 ${\text{cm}}^{-1}$ is due to the stretching vibration of the C=O bond^30^. The peak at 1568.2 ${\text{cm}}^{-1}$ corresponds to N–H bond^31,32^, while the peak at 1399.0 ${\text{cm}}^{-1}$ is due to the stretching vibration C–O–C^33,34^. These bonds are frequently detected on the surface of carbon dots.

**Fig. 4:**
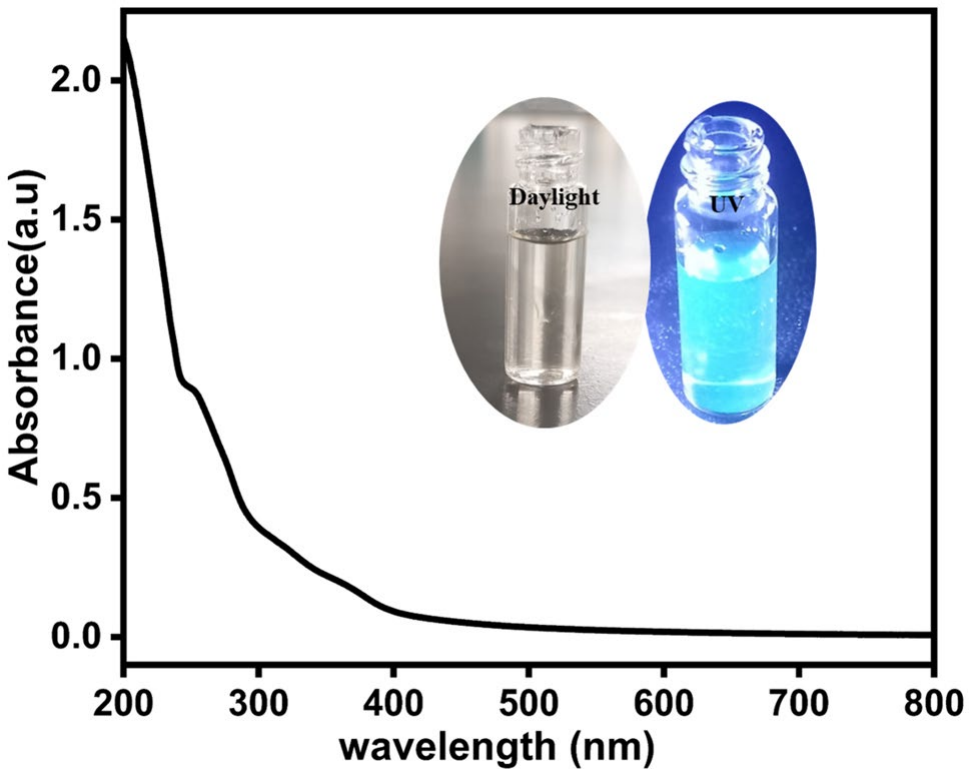
The absorption spectra of the synthesized carbon dots. The inset shows the CDs under daylight and UV illumination.

**Fig. 5:**
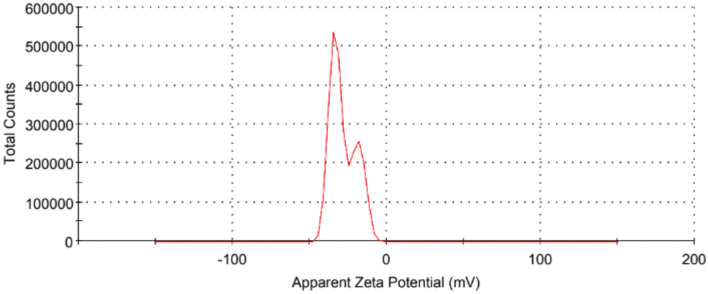
The Zeta potential diagram of the synthesized CDs.

UV absorption spectrum, Fig. 4, shows three peaks at 250.0 nm and 295 nm corresponding to the π–π* of C=C electron transition, and a peak at 366.0 nm for the n-π* electron transition^35,36^. The visible region of all CDs showed no significant absorption. This absorption spectra indicates that the electronic transition type of CDs is minimally influenced by the types of biomass carbon sources. It should be noted that our CDs show a bluish-green fluorescence under UV light illumination at 365.0 nm (see inset of Fig. 4). Moreover, the surface potential of the synthesized carbon dots was analyzed using Zeta potential analyzer at room temperature. Data shows excellent colloidal stability and dispersibility for the nanoparticles as the measured zeta potential value is − 32.9 mV, Fig. 5. Based on current knowledge, the measured zeta value for biomass-synthesized CDs is relatively high compared to other reported values.

**Fig. 6:**
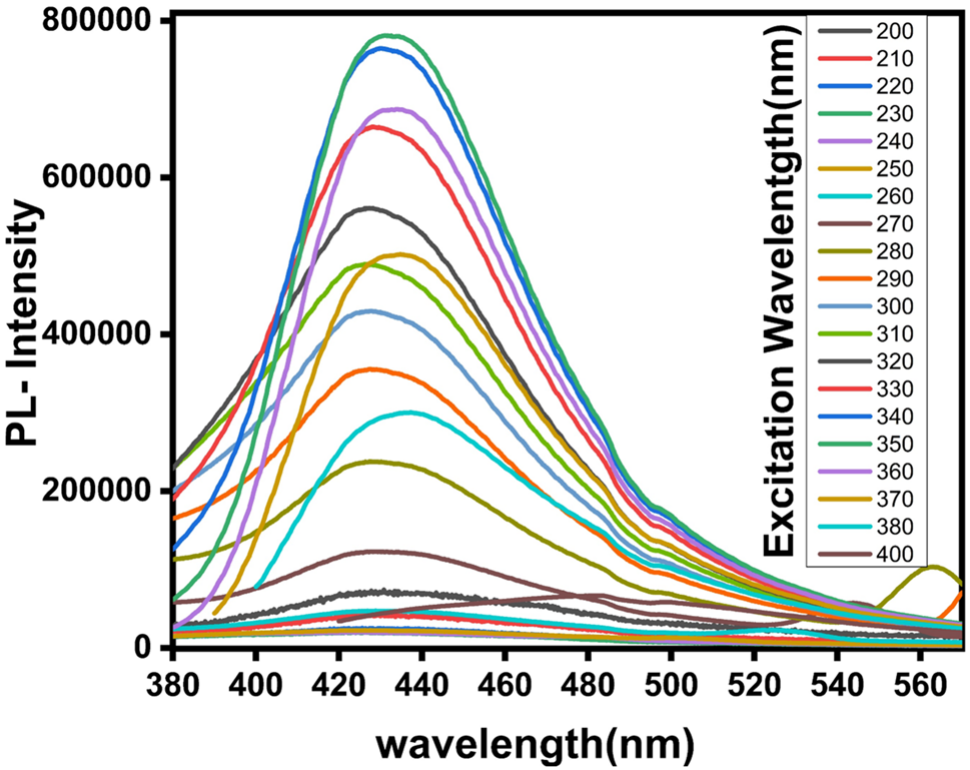
The photoluminescence spectra of the synthesized CDs under different excitation wavelengths in the range of 200–400 nm.

The Photoluminescence measurement, Fig. 6**,** showed that the sensitized carbon dot is excitation dependent. Namely, the fluorescent of the carbon dots exhibits a red shift, as the excitation wavelength increases. The maximum fluorescence intensity was found at 430 nm under excitation wavelength of 350 nm.

The PL-quantum yield (${\varvec{\phi}}$) of the prepared CDs was calculated using the following equation. ^37^

2 $${\phi }_{sample}= {\phi }_{reference}\left(\frac{{Gradient}_{sample}}{{Gradient}_{reference}} \times \frac{{\eta }_{sample}^{2}}{{\eta }_{reference}^{2}}\right)$$

**Fig. 7:**
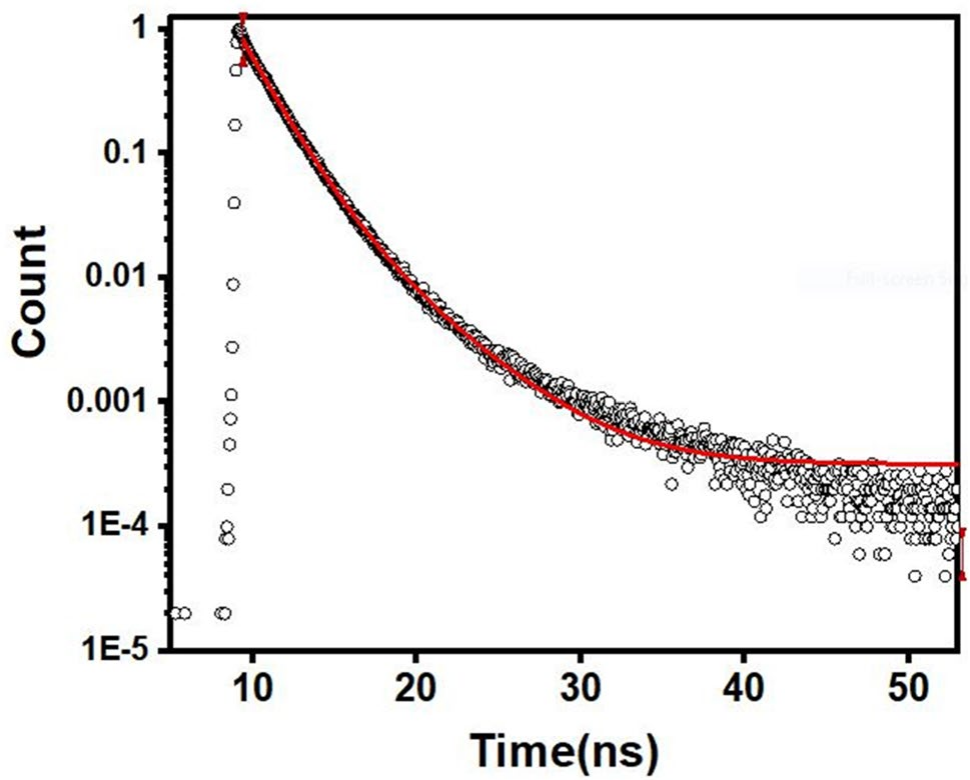
The photoluminescence lifetime decay of the synthesized CDs.

The gradient is the slope of the curve plotted between the integrated area of the fluorescence intensity (y-axis) and the absorbance (x-axis) at the excitation wavelength, while $\eta$ is the refractive index^37^. The calculated value of (${\varvec{\phi}}$) was 0.17% using quinine sulfate (= 0.54) as a reference. This characteristic is commonly observed and meets the requirements for sensing applications. Moreover. the average fluorescence lifetime of the obtained CDs was recorded to be 7.4 ns, as far as we know, it is uncommon for CDs derived from natural sources to exhibit such an extended lifespan, indicating that the dots we synthesized possess excellent optical characteristics, Fig. 7.

**Fig. 8:**
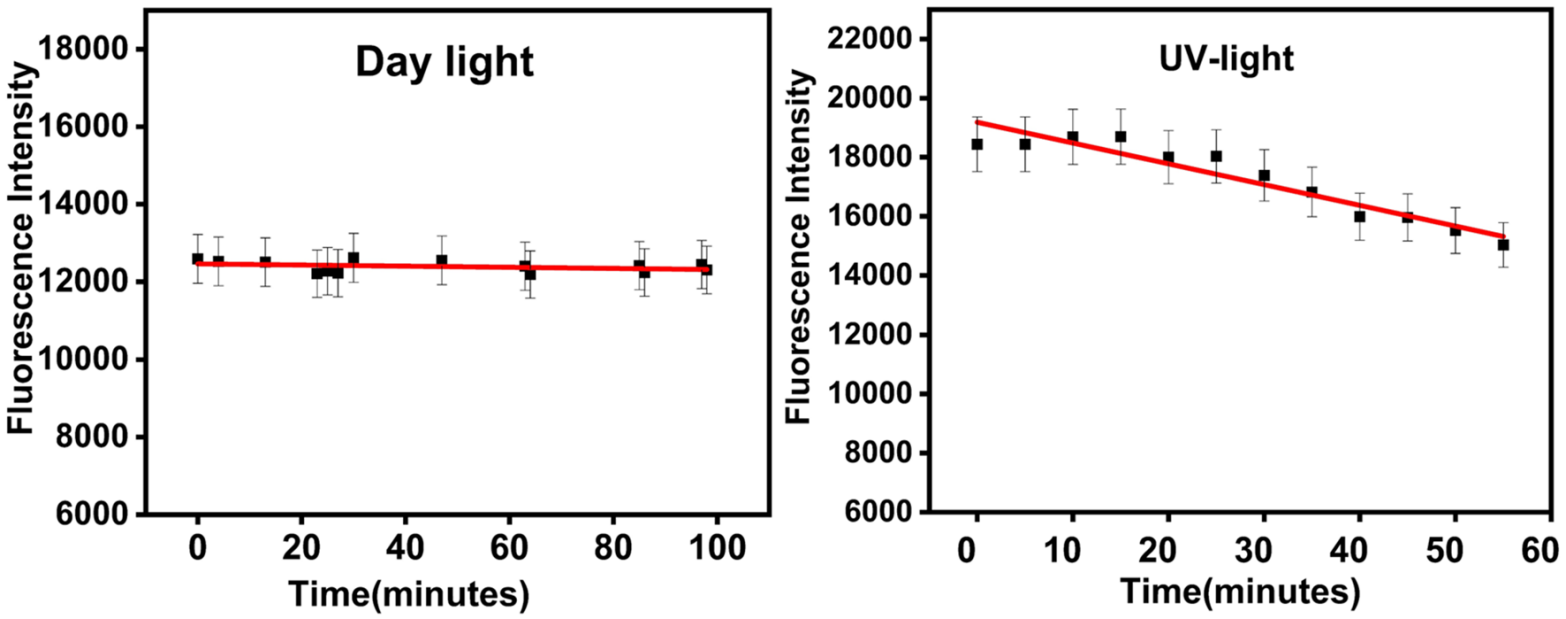
The photoluminescence stability of synthesized CDs under daylight and UV-illumination.

The fluorescence stability of the synthesized carbon dots was scrutinized over a 100-min duration under natural daylight and 60 min under UV light. The fluorescence data depicted in Fig. 8 substantiates the potential utility of these carbon dots as a sensor, given their remarkable photoluminescence stability during daylight exposure. However, it is noteworthy that the fluorescence of the specimen subjected to a 365 nm UV lamp declined after 20 min of exposure. Although this quenching phenomenon occurred, it is important to acknowledge that the 20-min duration remains considerably extended for a typical measurement timeframe for optical sensors.

### CDs as Kojic acid sensor

#### Linearity assessment

**Fig. 9:**
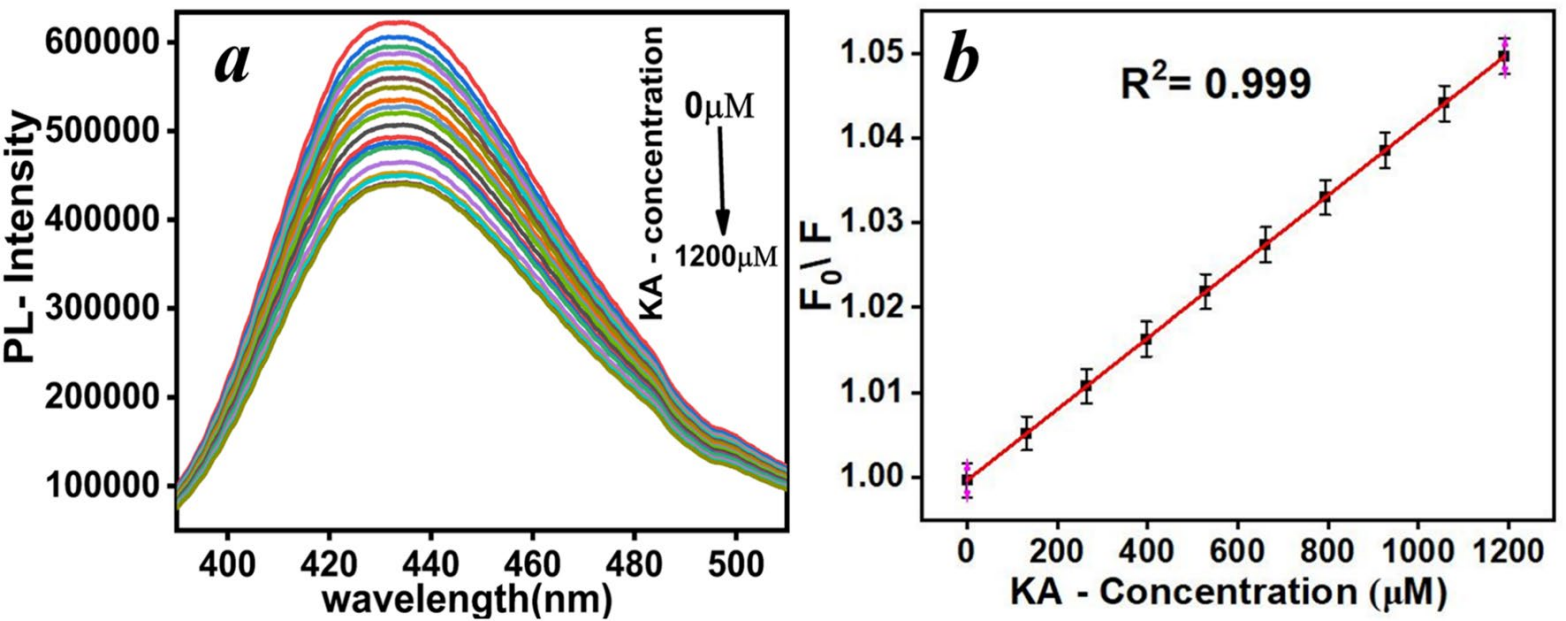
(**a**) The effect of different KA concentration (0–1200 µM) on the photoluminescence intensity of CDs and (**b**) the stern–Volmer plot.

KA quantitatively quenched the fluorescence intensity of the CDs at an excitation wavelength of 350 nm due to the interaction between the molecules. A Stern–Volmer plot, the measurement of F_0_/F against KA concentration, where *F*_0_ and *F* are the fluorescence intensity of the CDs in the absence and presence of KA, respectively, showed an excellent quantitative correlation (R^2^ = 0.999) within KA concentration range of 120–1200 µM. The limit of detection, LOD, was calculated to be 30 µM and LOQ is 90 µM, Fig. 9. The linear range offers a wide working range for the optical sensor to determine KA from 1200 µM down to 120 µM, which is suitable range for the real sample applications.

#### Selectivity assessment

**Fig. 10:**
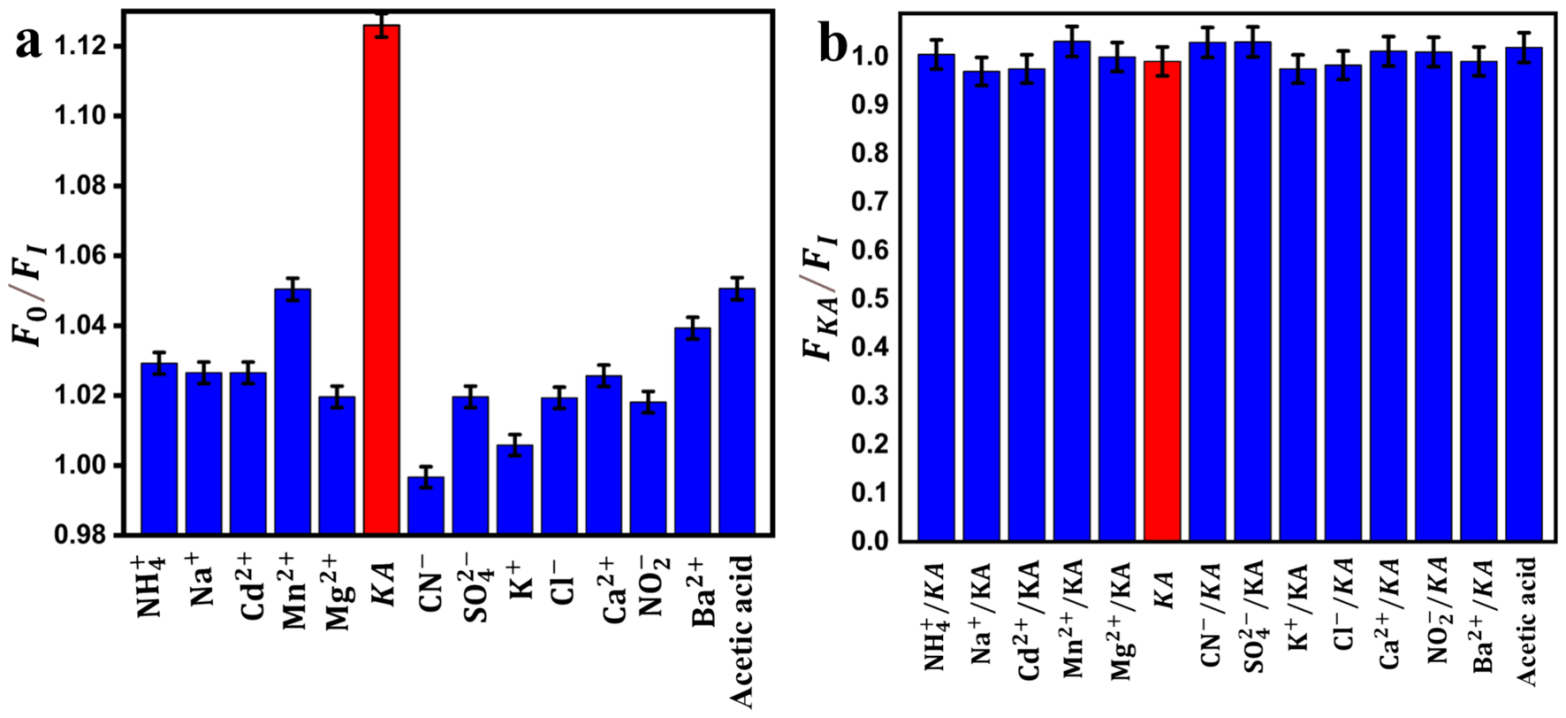
(**a**) The selectivity of CDs (F_0_/F) to KA against different common ions that may exist in food samples. (**b**) the effect of common ions on the fluorescence quenching of CDs in the presence of KA (the concentration of other analytes is ten-fold(2500 μM) that of KA (250 μM).

Selectivity is a mandatory sensor performance parameter, especially for real sample applications. In this work selectivity study was performed over two levels. The first Level studies the quenching effect of KA compared to common interferents on the fluorescent carbon dots. While level 2 studies the interference exerted by the common interferents on the KA-CDs interaction. The selectivity experiment was done in the presence of a ten-fold concentration of the potential interferents. Figure 10a shows a compatible quenching response of KA compared to that induced by ten times the KA concentration of any of the studied interferents, which indicates higher sensor selectivity towards KA. Results represented in Fig. 10b prove a stable fluorescence quenching in the presence of the studies interferents.

#### Quenching mechanism

**Fig. 11:**
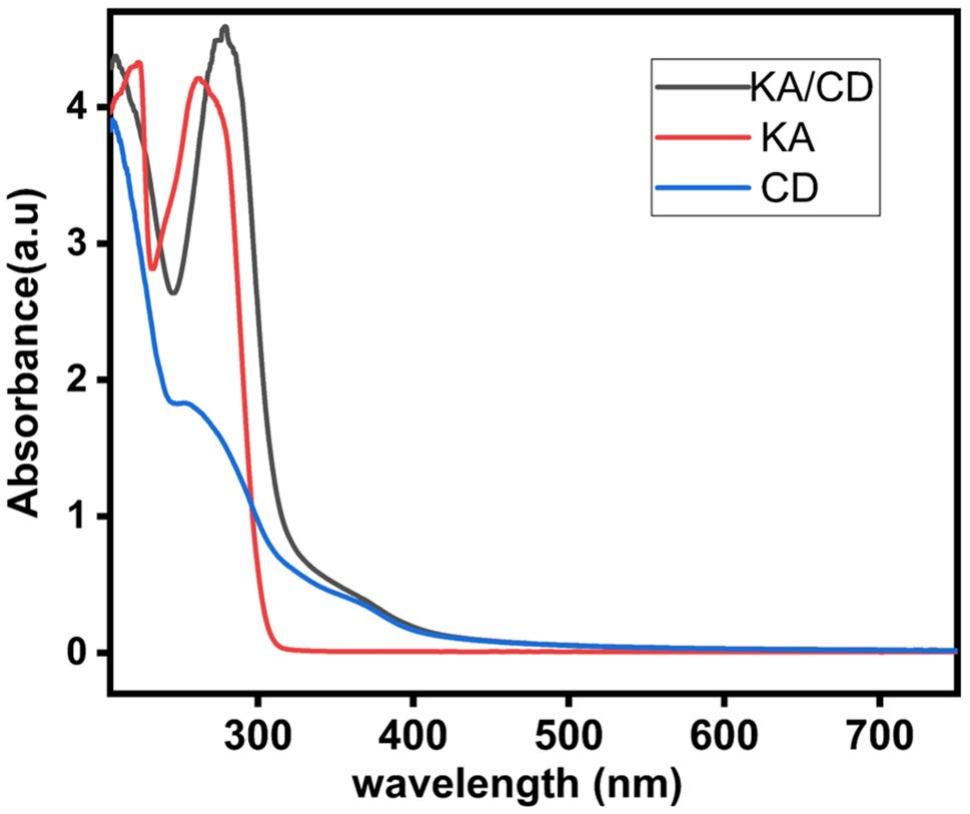
The UV–Vis absorbance of KA, CD, and KA/CD solutions.

To demonstrate how KA affects the fluorescence of CDs. The mechanism occurs through a static quenching process, while the fluorescence quenching mechanism of CDs results from the formation of a non-fluorescent complex of CD and KA due to the interaction between the fluorophore and the quencher in the ground state. When this complex absorbs exciting wavelength, it promptly returns to the ground state without emitting radiative photons through non-radiative deactivation mechanisms^38^. This effect was confirmed by analyzing the UV–Vis absorption spectra of CDs, KA (quencher), and KA/CD mixture solution. Notably, a significant red shift of 13 nm and 15 nm was observed in the two characteristic peaks of KA. Moreover, the emergence of a broad peak between 350 and 380 nm, which overlapped with the excitation wavelength, led to an inner filter effect, Fig. 11.

**Fig. 12:**
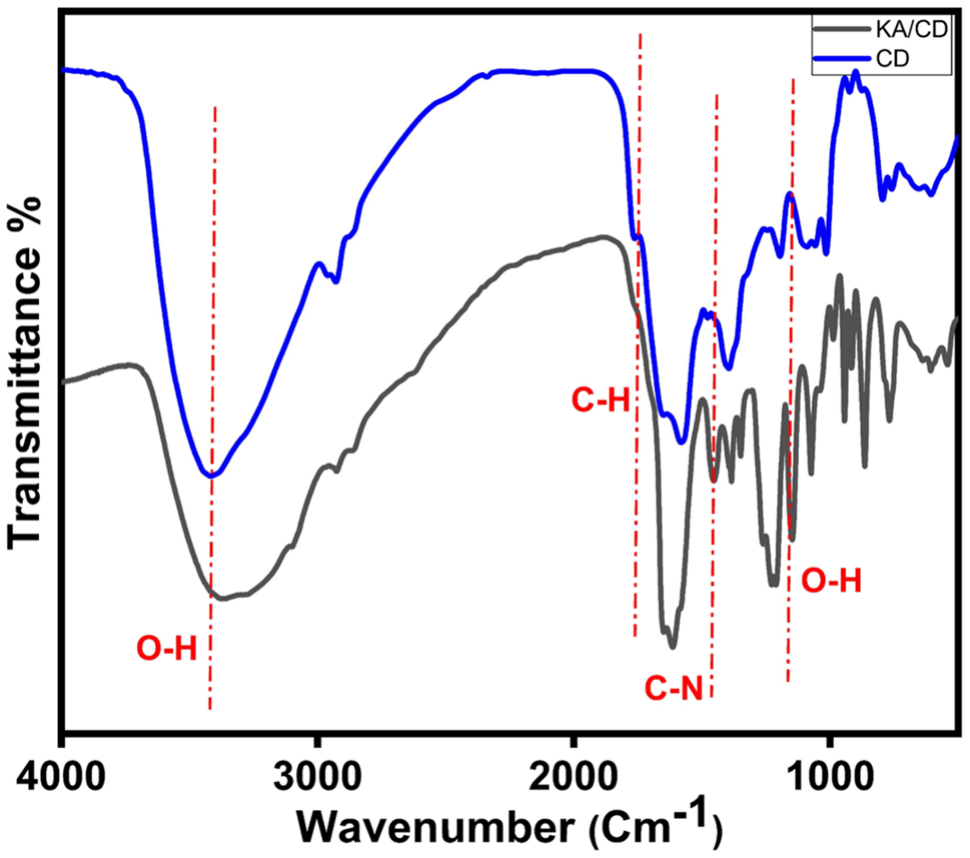
FT-IR spectrum of synthesized CDs and CDs/KA*.*

The IR spectra of CDs, KA, and KA/CDs mixture confirm the formation availability of a new complex due to the existence of hydrogen bonds. In a hydrogen bond, the hydrogen atom acts as a bridge between two electronegative atoms, resulting in a relatively strong dipole–dipole interaction. This bond is characterized by electrostatic attraction between the partially positive hydrogen atom and the partially negative atom it is bonded to. IR spectroscopy analyzes the stretching and bending vibrations of the atomic bonds involved to identify the presence of hydrogen bonds. Typically, hydrogen bonding causes a shift or broadening of relevant peaks in the IR spectrum^39^. The change in the IR spectra of the CDs functional groups, namely, C=C, C=O, and N–H, indicates KA/CD interaction. The O–H peak at 3420.08 ${\text{cm}}^{-1}$ exhibited broadening due to the formation of new hydrogen bonds, the C–H bond peak at 1757.32 ${\text{cm}}^{-1}$ disappeared, while new peaks emerged at 1145.40 ${\text{cm}}^{-1}$ represented the O–H bond, and a peak emerged at 1450.00 ${\text{cm}}^{-1}$ for C–N bond. The O–H and C=O functional groups of KA establish van der Waal forces and hydrogen bonds with carbon dots, Fig. 12.^40^ These notable alterations provide insights into how the addition of kojic acid impacts the structure of carbon dots (CDs), ultimately resulting in the molecule returning to its ground state through non-radiative emission.

### Comparison of performance between current method and the reported methods

Table 1 gives a comparison between our method and other existing methods in literature. As seen our method is simpler to use and has a wider range for accurately measuring the concentration of kojic acid compared to other methods in literature. This is particularly important because kojic acid is commonly used as a food additive, and our method covers the desired concentration range for this purpose. In addition, our approach has notable benefits in terms of being environmentally friendly, and cost-effective.

**Table 1:** KA detection using different methods in the literature.

Method	Linear material	Range (μM)	LOD (μM)	Reference
Electrochemical	*o*-phenylenediamine as the functional monomer	0.01–0.2	0.003	^41^
Electrochemical	Ti3C2 MXene, Prussian blue, and gold nanoparticles	1–600	1.0	^42^
Electrochemical	carbon paste electrode modified with 1-butyl-3-methylimidazolium tetrafluoroborate	5.0–600	0.8	^43^
Magnetic solid phase extraction (MSPE) adsorbent	Fe_3_O_4_@MIP	0.35–3518.40	0.15	^44^
Fluorescence	gold nanoclusters (AuNCs)	10–1000	5	^6^
HPLC	–	140–703	1.4	^45^
Electrochemical	multi-walled carbon nanotubes	20–5000	16	^46^
Fluorescence	Copper nanocluster	0.2–50	0.07	^7^
Fluorescence	Carbon dots	120–1200	30	Our work

### Kojic acid in real samples

Certain food samples like soy sauce and vinegar may contain a small quantity of KA that may intentionally be added to the products for preservation purposes. This has a health impact on the consumers. The performance of the CDs sensor was evaluated by assessing the recovery percentage and relative error as measures of accuracy using the standard addition method as a method of validation, in which each single Standard KA addition experiment was performed three times^47^. The estimation of KA concentration in the soy sauce sample was found to be 523 μM, and the corresponding results are presented in Table.2 The average recoveries of the added standard KA solution is calculated to be 103.5%, and with relative standard deviation (RSD%) values with average value of 1.04%. In the case of the vinegar sample, the estimated concentration of KA was 508 μM (Table 2). The recovery for vinegar is within an average of 106%, with an RSD% average value of 2.525.

**Table 2:** Determination of the kojic acid in soy sauce and vinegar samples.

Sample	Found KA* (µM)	Standard KA added (µM)	Standard KA Found (µM)	Standard KA Recovery ± RSD* (%)
Soy sauce sample	525.67 ± 0.79	609.75	646.91	106.09 ± 1.63
		1190.40	1202.10	100.98 ± 0.63
		1704.54	1755.34	102.98 ± 0.45
		2222.22	2275.03	102.38 ± 1.11
Average				103.11 ± 0.96
Vinegar sample	508.04 ± 1.60	609.75	662.47	108.65 ± 4.47
		1190.47	1222.44	102.69 ± 2.21
		1704.54	1762.86	103.42 ± 1.27
		2222.22	2289.54	103.03 ± 0.64
Average				104.45 ± 2.15

*Average of three determinations (n = 3).

## Conclusion

In conclusion, this study introduces an innovative method for detecting kojic acid (KA) using carbon dots synthesized from pomegranate peel powder. This approach is environmentally friendly, cost-effective, and straightforward. The resulting carbon dots serve as a highly sensitive, linear, and selective optical sensor for KA detection, with impressive performance metrics including a high linearity (R^2^ = 0.999) across a range of KA concentrations (120 μM to 1200 μM) and a low detection limit (LOD = 30 ± 0.04 μM). The sensor's effectiveness was validated through selectivity tests and real sample measurements, demonstrating its potential for evaluating food quality. This research highlights the promising future of carbon dots from pomegranate peel in KA detection and suggests novel prospects for practical applications in various industries.

## Supplementary Information

Supplementary Information.

## Data Availability

The datasets used and/or analyzed during the current study available from the corresponding author on reasonable request.
