# Supplementary material for: Highly sensitive detection of kojic acid in food samples using fluorescent carbon dots derived from pomegranate peel
Source: Sci Rep. 2024 Sep 10;14:21144. doi: 10.1038/s41598-024-70844-2 (PMC11387480; doi:10.1038/s41598-024-70844-2)
Supplement: Supplementary file 1 — Supplementary Information. [file 41598_2024_70844_MOESM1_ESM.docx]

**supplementary information**

**Provided below lifetime data from with fitting that processed by the software:**

**
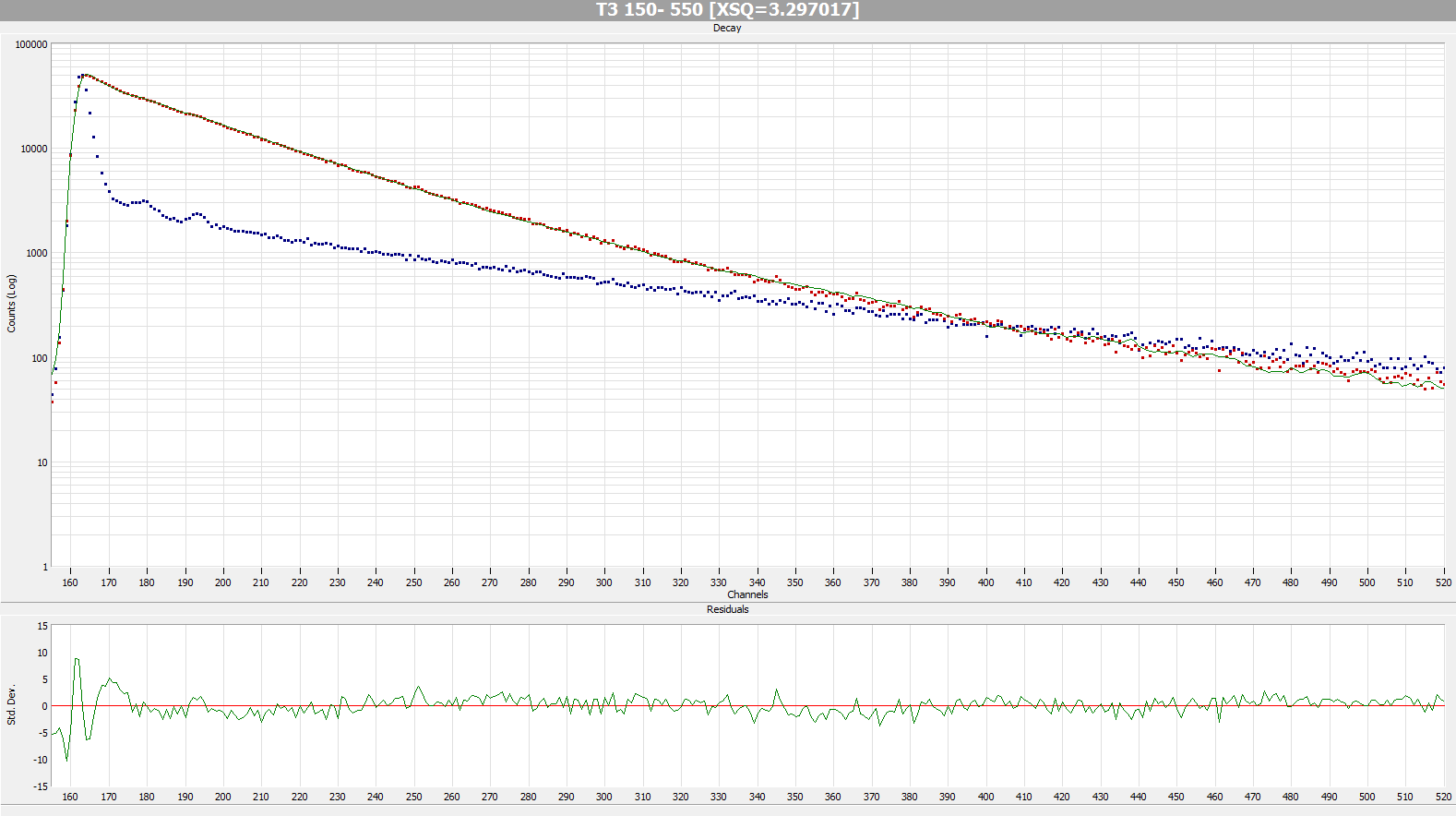
**

Figure 1. The photoluminescence lifetime decay of the synthesized CDs from the software data


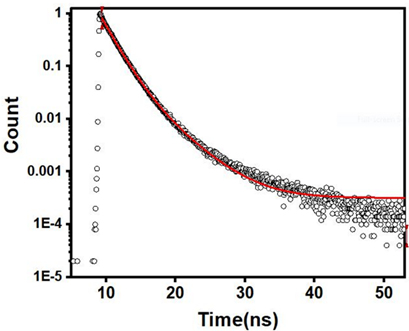


Figure 2. The photoluminescence lifetime decay of the synthesized CDs plotted from raw data obtained from device.

The average lifetime calculated by software that uses the following equations of lifetime

$I_{\left( t \right)}=\sum_{i}^{n} \alpha_{i}e^{(-\frac{t}{\tau_{i}})}$ [Equation 1]

where *t* is time, *τ* is the fluorescence lifetime, and *B* is the pre-exponential factor. The fluorescence lifetime is defined as the time it takes the intensity to drop to 1/e (=0.368) of its initial value.

The fit can be evaluated by calculation of the $ꭓ^{2}$. This function calculates the difference between the raw data points and the fitting points to quantify how well the data has been replicated.

$ꭓ^{2}=\sum_{K=1}^{n} \frac{{[N\left( t_{k} \right)-N_{C}\left( t_{k} \right)]}^{2}}{N\left( t_{k} \right)}$ [Equation 2]

The difference between the measured fluorescence decay function, [Equation1], and the calculated decay function, [Equation 2], are evaluated across the number of data points, n.

$ꭓ^{2}$ value of 1 indicates that the system is replicated by the fit. A value above 1.2 can indicate that the system is not well described by the fit. The bounds for an acceptable $ꭓ^{2}$ will vary on the experimental system, specifically with the noise associated with it. Good practice should include measurement of a well-established single component fluorophore prior to sample measurement to establish the achievable sensitivity of the system.
